# Plastids in a Pinch: Coordinating Stress and Developmental Responses Through Retrograde Signalling

**DOI:** 10.1111/pce.15664

**Published:** 2025-06-05

**Authors:** Elizabeth van Veen, Jesse J. Küpers, Charlotte M. M. Gommers

**Affiliations:** ^1^ Laboratory of Plant Physiology Wageningen University and Research the Netherlands

**Keywords:** abiotic stress, chloroplasts, environmental adaptation, gene expression regulation, genomes uncoupled, photosynthesis, plant development, retrograde signalling

## Abstract

Plastids are crucial for fuelling and regulating plant growth and development. Photosynthesising chloroplasts provide energy for growth, while other plastids play additional key roles in various aspects of plant physiology. For function and development, plastids greatly depend on nucleus‐encoded proteins, and they can modulate the synthesis of these proteins by sending retrograde signals to the nucleus. These signals communicate the developmental and operational status of the plastid, both of which are sensitive to the environment. Abiotic stressors such as drought, salinity, and suboptimal light and temperature conditions can induce changes in chloroplast metabolism, ultrastructure and cellular positioning. In response to specific environmental triggers, retrograde signals reprogramme nuclear gene expression to fine‐tune plastid form and function, but also influence whole‐plant morphology. Over the past years, the chloroplast responses to stress have become clearer. Various sources of retrograde signals, derived from plastid metabolism, plastid gene expression and altered photosynthetic redox balance, are now known to directly interfere with canonical signalling pathways. However, most of what is known about retrograde signalling originates from studies using artificial stressors, such as chemical treatments or genetic mutations, and its importance in natural environments is still poorly understood. This review highlights the understanding of plastid responses to the environment, as well as the impact generated downstream of retrograde signals, to better understand the role of plastids in abiotic stress resilience of flowering plants.

## Summary statement

Plastids can sense environmental stress and improve whole plant adaptation through retrograde communication to the nucleus. Recent studies have advanced our understanding of integrated canonical and retrograde signalling during abiotic stress, which fine‐tune gene expression and development.

## 1 Introduction

Plant life as we know it depends on plastids, whether it be for photosynthesis, producing essential metabolites or even steering plant development. Plastids, such as chloroplasts, are generally believed to have evolved during a single endosymbiotic event, where a free‐living photosynthetic cyanobacterium was engulfed by an ancestral eukaryotic host. The transition from free‐living microbes to organelles involved a drastic reduction of genome size, which nowadays amounts to only 5%–10% of that in free‐living cyanobacteria (Martin et al. 2002). The remaining plastid genome, the plastome, encodes anywhere from 120 genes in plants, up to approximately 200 genes in certain red algae (Pfannschmidt et al. 2015). The dependency of plastids on nuclear transcription allows the host cell to steer plastid development, resulting in a range of different plastid types.

Both the development and functionality of plastids are sensitive to environmental signals. For example, in newly germinated seedlings, etioplast‐to‐chloroplast biogenesis depends on the presence of light (Pipitone et al. 2021). For mature chloroplasts, any environmental signal that impacts photosynthesis has concomitant effects on its functionality, including not only light but also salinity, temperature and drought.

For plants to succeed in a dynamic natural environment, chloroplast development and operation must be plastic. Having said that, chloroplasts cannot operate alone, but rather in conjunction with the nucleus. Additionally, alterations in plastid metabolism can have significant effects on the host cell. To coordinate the coupling of chloroplast well‐being and nuclear gene expression, signalling systems communicate from the plastids to the nucleus (via retrograde signals) and vice versa (via anterograde signals) (Hernández‐Verdeja et al. 2020). Significant progress has been made in deducing the nature of chloroplast‐derived retrograde signals (reviewed in [Chan et al. 2016b]). Notwithstanding these findings, the majority of these studies relied on the use of chemical stressors or genetic mutations that induce chloroplast malfunction. The contribution of chloroplast signals to more natural stress responses and plant development remains to be comprehensively reviewed. Here, we summarise the latest developments in the understanding of how chloroplasts are affected by abiotic stress, how this steers chloroplast‐derived retrograde signals and how these contribute to whole‐plant acclimation to the environment. Despite our efforts to include all recent developments, we apologise to those whose work could not be added due to space restrictions.

## 2 The Impact of Abiotic Stress on Chloroplast Form and Functionality

Plants are continuously exposed to fluctuating and possibly detrimental environmental conditions that may affect cellular homoeostasis by inducing reactive oxygen species (ROS), limiting water availability or altering metabolic processes. Such stress‐induced cellular changes can affect various aspects of chloroplast form and functionality.

### 2.1 Chloroplast Ultrastructure and Positioning

**Figure 1:**
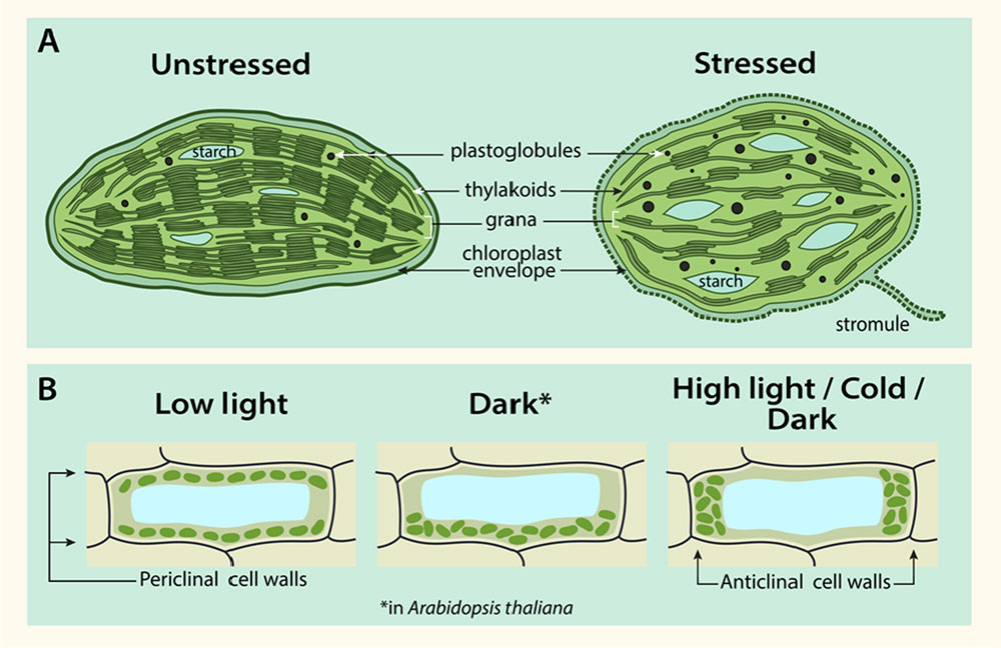
Abiotic stressors affect chloroplast ultrastructure (A) and chloroplast positioning within the cell (B). Chloroplast ultrastructure changes under stress include rounding of the chloroplast, increased plastoglobuli size and number, stromule formation, dismantlement of the thylakoid network, grana unstacking, altered numbers of starch granules and chloroplast envelope damage (as indicated by dashed line). (B) Chloroplast positioning is altered under different light and temperature conditions. Under low light, chloroplasts orient along periclinal cell walls, but under high light, they orient along anticlinal cell walls. Positioning in the dark conditions differs between species, with the chloroplast moving to the bottom of the cell in *Arabidopsis*, but aligning along the anticlinal cell walls in other species. In response to cold under low‐light conditions, chloroplasts also move to the anticlinal cell walls. Representative cells show a side view.

Abiotic stresses can induce changes in chloroplast ultrastructure such as reduced grana stacking, swelling of the thylakoid lumen and a loss of thylakoid organisation (Wang et al. 2014; Wang et al. 2019). Thylakoid disassembly leads to the mobilisation of lipophilic compounds that accumulate in lipid reservoirs, the plastoglubules. These plastoglobules increase in number and size in response to stress conditions (Wang et al. 2019; Zhang et al. 2010). Additionally, abiotic stressors can compromise chloroplast envelope membrane integrity, which is associated with increased stromule formation (Figure 1A) (Gray et al. 2012). While their function hasn't been fully resolved, stromules have been suggested to be involved in inter‐organellar communication.

While the aforementioned responses to stress are relatively consistent, studies have yielded inconsistent findings related to the presence of starch granules following stress treatment. Chilling stress causes both a decrease and an accumulation of starch granules. The decrease has been attributed to either a reduction in photosynthesis rate or the breakdown of starch granules to facilitate the biosynthesis of osmoprotectants (Ribeiro et al. 2022). Other studies have attributed an accumulation of starch granules in response to decreased respiration and starch hydrolysis rates following cold exposure (Zhuang et al. 2019). This disparity in findings likely reflects a dynamic response to stress conditions in terms of starch metabolism (Ristic and Ashworth 1993).

The shape and positioning of chloroplasts within the cell vary substantially between stress and non‐stress conditions. Chloroplasts often become more spherical or thicker in stress (Meng et al. 2016; Zhang et al. 2010) (Figure 1A). Additionally, chloroplasts alter their positioning under different environmental conditions so as to optimise light absorption for photosynthesis and avoid damage to the photosynthetic machinery (Figure 1B). These movements have been best studied in the context of light, where chloroplasts position themselves along the anticlinal (side) cell walls under potentially harmful high light intensities, but accumulate in layers along the periclinal (top and bottom) cell walls under dim light (Schramma et al. 2023; Davis and Hangarter 2012). Interestingly, under dark conditions, chloroplasts accumulate along the bottom of cells in *Arabidopsis* (Suetsugu et al. 2005), but along the anticlinal walls in non‐angiosperms such as the liverwort *Apopellia endiviifolia* (Yong et al. 2021) (Figure 1B).

Temperature stress also alters chloroplast positioning. For example, in dim light at low temperatures (approximately 5°C), chloroplasts orient along the anticlinal cell walls (Ogasawara et al. 2013), likely as a means to reduce cold‐induced photosystem II (PSII) photoinhibition. The movement of chloroplasts in response to light and temperature depends on the blue light photoreceptor phototropin (Fujii et al. 2017). In addition to light and temperature, abiotic stressors, including drought and salinity, can alter chloroplast positioning (Yamada et al. 2009). Although chloroplast movements under these stresses likely play a role in photoprotection or the maintenance of photosynthesis as well, their function is yet to be fully elucidated.

### 2.2 Chloroplast Functionality: Photosynthesis

The photosynthetic system is incredibly sensitive to fluctuating environmental conditions. Many of the earlier‐mentioned changes in chloroplast ultrastructure reflect this. Additionally, abiotic stresses can impose rapid disruptions to photosynthesis by affecting enzyme activity, redox homoeostasis and pigment biosynthesis and directly damaging the photosystems. The impact on photosynthesis, however, can vary considerably depending on the nature and intensity of the stress. Here we summarise the effects of drought, salinity, heat stress and high/low light conditions.

Drought markedly reduces photosynthetic efficiency by a number of mechanisms. In response to drought, plants typically close their stomata and reduce transpiration to conserve water reserves (Sperry et al. 2017). This entails a trade‐off whereby CO_2_ intake is reduced, which slows the Calvin cycle and limits NADP^+^ availability to the electron transport chain (Noctor et al. 2014). Additionally, excess O_2_, resulting from continued photosynthesis and closed stomata, can induce the wasteful process of photorespiration, which concurrently acts as a source of oxidative stress (Noctor 2002). At a biochemical level, drought leads to over‐reduction of the electron transport chain (Noctor et al. 2014), which can cause over‐excitation of the PSII reaction centre. Excess energy may be passed on to O_2_ (which becomes enriched when stomata are closed) and yield singlet oxygen (Dmitrieva et al. 2020). Additionally, O_2_ may act as an alternative electron acceptor, being reduced to superoxide (O_2_^−^), and subsequently hydrogen peroxide (H_2_O_2_) and the hydroxyl radical (^•^OH), which induces oxidative stress (Gururani et al. 2015). These ROS may further decrease photosynthetic efficiently by directly damaging the D1 and D2 proteins of PSII (Krieger‐Liszkay 2005), disrupting photosynthetic pigments (Shin et al. 2021), as well as inducing lipid peroxidation of thylakoid membranes (Killi et al. 2020). The damage to PSII further reduces its quantum yield efficiency through photoinhibition (Moustakas et al. 2022). To counteract these deleterious effects, plants have evolved photoprotective mechanisms such as antioxidant enzymes (Logan et al. 2006), PSII damage repair (Theis and Schroda 2016), non‐photochemical quenching (Lu et al. 2022) and PSI cyclic electron flow (Huang et al. 2018). The balance between damage imposed by abiotic stressors and the photoprotective potential within the chloroplast dictates the harmfulness to plant productivity.

Photosynthetic damage imposed by drought also occurs under salt stress. Even so, the toxic effects of sodium (Na^+^) and chloride (Cl^−^) ions add to the osmotic effects. For example, high ion concentrations affect plastid membrane stability (Wang et al. 2014). The uptake of Na^+^ into the cell via non‐selective cation channels induces cellular electrolyte leakage, which results in a dramatic loss in cellular K^+^ levels, which inhibits photosynthesis at multiple levels (Van Zelm et al. 2020).

The impact of ionic stress induced by salinity is difficult to study independently of the accompanying osmotic stress without using proper controls. The detrimental effects of Na^+^ on photosynthesis, however, have been exhibited through the use of Na^+^ channel blockers, which can partially rescue the photosynthetic perturbations inflicted by salinity (Zhang and Xing 2008). On a similar note, overexpression of the *Arabidopsis* vacuolar Na^+^/H^+^ antiporter *NHX1 (Sodium/Hydrogen Exchanger 1)* improved photosynthesis via Na^+^ sequestration (He et al. 2005). Intriguingly, while ion toxicity is typically attributed to Na^+^ ions, Chen and Yu (2007) found inhibition of photosynthesis in soybean to be impacted by Cl^−^ rather than Na^+^ ions. Interestingly, some halophytic species require high Cl^−^ concentrations to maintain optimal photosynthesis (Preston and Pace 1985), which suggests that many of these responses differ between species.

Heat stress affects photosynthesis at different levels. First of all, in most plant species, warm temperatures cause a reduction in photosynthetic pigments (chlorophylls and carotenoids), which results in reduced light harvesting and should prevent ROS accumulation (Jespersen et al. 2016). Evergreen grasses that lack a Chla catabolic protein accumulate more ROS and experience severe photodamage when exposed to heat stress (Zhang et al. 2022). Heat causes dismantling of PSII–LHCII complexes and the degradation of many PSII‐ and PSI‐associated proteins, leading to reduced photosynthetic activity (Zhang et al. 2022). In addition, carbon fixation is strongly impacted by heat stress, due to slower reactivation of Rubisco by activase enzymes (Salvucci and Crafts‐Brandner 2004).

One other abiotic factor that affects chloroplast primary metabolism is light. Different aspects of light can impact photosynthesis, including fluctuations in light intensity (Morales and Kaiser 2020) and spectral composition (Liu and Van Iersel 2021). Akin to drought stress, a sudden increase in light intensity can induce over‐reduction of the photosynthetic electron transport chain (Yamori et al. 2016), which inevitably results in ROS formation and photoinhibition (Shi et al. 2022). The aforementioned photoprotective mechanisms come into play to maintain redox homoeostasis under these over‐reducing conditions. In addition, maintaining photosynthesis under high light stress requires repair of PSII (Shi et al. 2022).

## 3 Plastid‐Derived Retrograde Signals

Given the limited capacity for self‐regulation via the plastid genome, the ability of chloroplasts to adapt to local conditions requires tight communication with the nucleus. Chloroplast‐to‐nucleus retrograde signals can be categorised at either biogenic or operational control levels. ‘Biogenic control’ refers to signalling that occurs during plastid development, whereas ‘operational control’ refers to signals released by mature chloroplasts in response to environmental fluctuations or stress (Chan et al. 2016b). The retrograde signalling pathways annotated to date are complex, and many signals remain to be fully described. Nevertheless, four major categories of signals exist: (1) intermediates of the tetrapyrrole biosynthesis pathway; (2) signals from altered plastid gene expression (PGE); (3) photosynthesis‐derived signals arising from altered thylakoid redox homoeostasis and (4) signals derived from other metabolic pathways (Figure 3). In the following sections, we will review the recent understanding of how these different classes of plastid‐derived signals act as retrograde signals.

### 3.1 Coupling of Genomes and the Tetrapyrrole Biosynthesis Pathway

The discovery that *Photosynthesis Associated Nuclear Gene* (*PhANG*) expression relies on functional chloroplasts provided insights into the biogenic control of retrograde signals. Forward genetic screens in *Arabidopsis* using the carotenoid biosynthesis inhibiting herbicide norflurazon led to the identification of the *gun* (*genomes uncoupled*) mutants. Exogenous treatment of light‐exposed plants with norflurazon typically triggers a strong repression of *PhANGs*. The *gun* mutants fail to exhibit this response, suggesting an impairment in chloroplast‐to‐nucleus communication (Susek et al. 1993; Mochizuki et al. 2001; Adhikari et al. 2011). Notably, five of the six *GUN* genes/alleles (*GUN2–GUN6*) directly regulate the tetrapyrrole synthesis pathway, thereby presenting this pathway as a source of biogenic retrograde signals.

**Figure 2:**
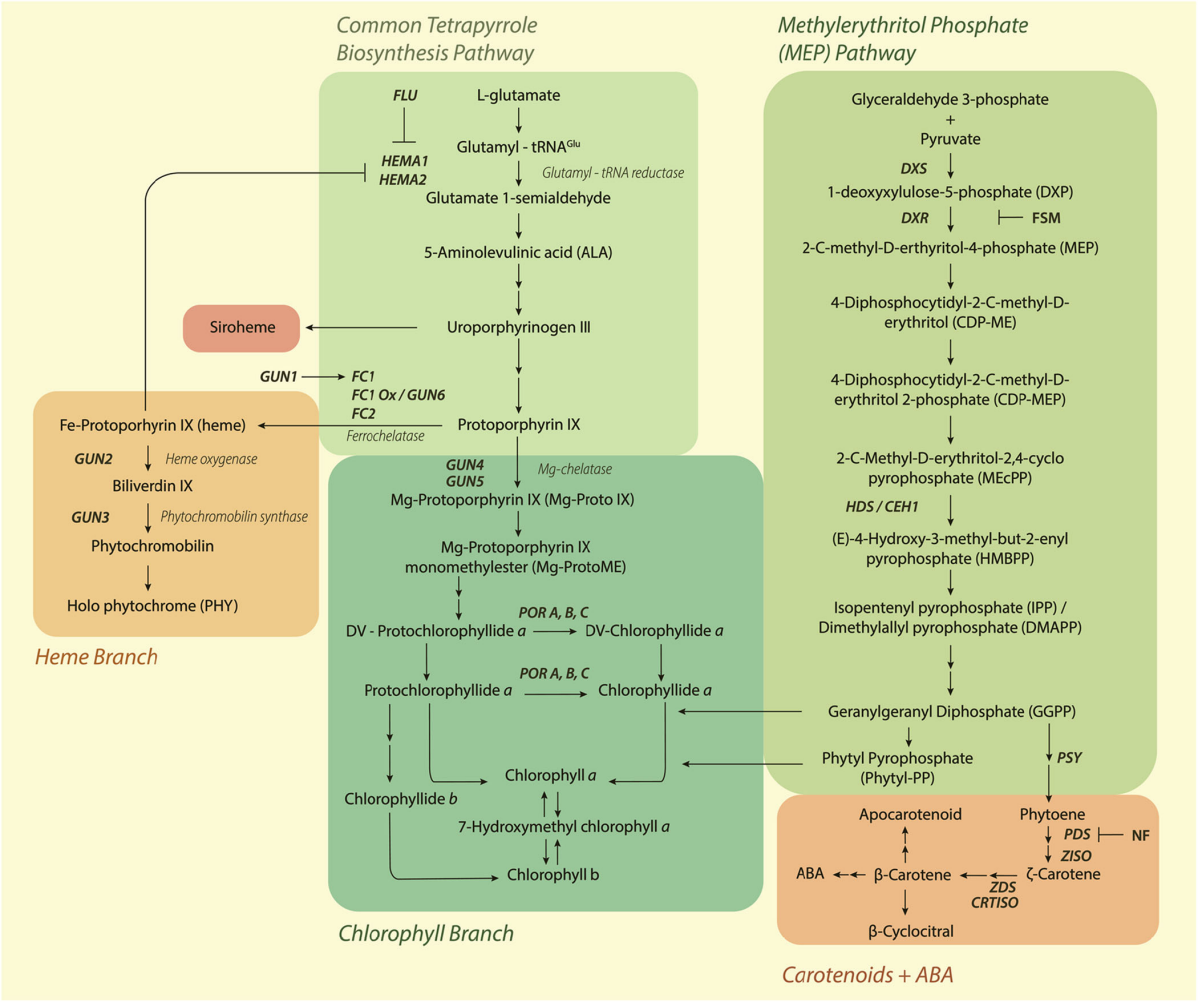
Simplified model for tetrapyrrole biosynthesis, the methylerythritol phosphate pathway and carotenoid biosynthesis pathways in plants. Single arrows indicate direct steps, double arrows indicate indirect steps, enzymes are in italics, and gene names are in bold italics. Boxes correspond to labels directly above/below. Abbreviations: ABA abscisic acid, *CEH1 Constitutively Expressing HYDROPEROXIDE LYASE 1*, *CRTISO CAROTENOID ISOMERASE*, *DXR 1‐DeoxyXylulose‐5‐phosphate Reductoisomerase*, *DXS 1‐DeoxyXylulose‐5‐phosphate Synthase*, *FLU FLUORESCENT IN BLUE LIGHT*, FSM fosmidomycin, *GUN GENOMES UNCOUPLED*, *HEMA HEME A*, NF norflurazon, *PDS phytoene desaturase*, *POR protochlorophyllide oxidoreductase, PSY phytoene synthase*, *ZDS ζ ‐carotene desaturase*. [Color figure can be viewed at wileyonlinelibrary.com]

*GUN2* and *GUN3* encode the enzymes haem oxygenase and protochromobilin synthase, respectively, and mutations in these genes lead to haem accumulation (Mochizuki et al. 2001). *gun6* also alters haem, but by encoding a gain‐of‐function allele for *FERROCHELATASE1*, which directly synthesises haem from protoporphyrin IX (Woodson et al. 2011) (Figure 2). Contrastingly, *GUN4* and *GUN5* are involved in synthesising the first committed chlorophyll precursor, Mg‐protoporphyrin IX (Mg‐ProtoIX) (Adhikari et al. 2011). *GUN5* encodes the H‐subunit of Mg‐chelatase, while *GUN4* encodes a Mg‐chelatase activator that binds its H‐subunit, substrate and product (Mochizuki et al. 2001; Larkin et al. 2003) (Figure 2).

**Figure 3:**
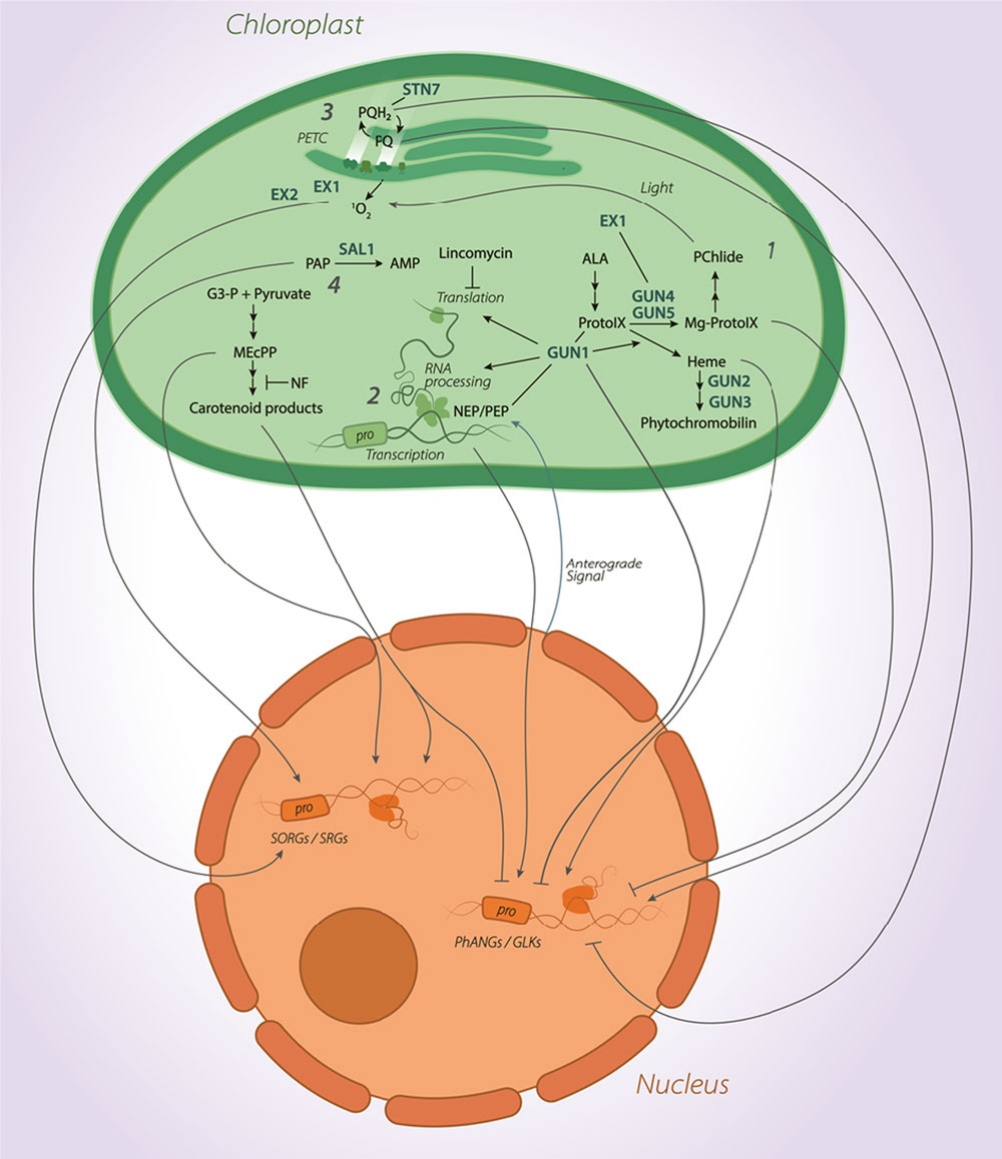
Simplified schematic representing four major retrograde signalling sources: (1) intermediates of the tetrapyrrole biosynthesis pathway, (2) signals from altered plastid gene expression, (3) photosynthesis‐derived signals and (4) signals arising from other metabolic pathways. Grey arrows indicate retrograde signals with either promotion (arrowhead) or repression (block arrow) of *Singlet Oxygen Responsive Genes/Stress Responsive Genes* or *Photosynthesis Associated Nuclear Genes/Golden Like* transcription factors. The chloroplast is shown in green, and the nucleus is shown in orange. [Color figure can be viewed at wileyonlinelibrary.com]

Following the identification of the *gun* mutants, researchers set out to determine whether the tetrapyrrole intermediates encoded by these genes act as mobile retrograde signals. Of these, the porphyrin intermediates Mg‐ProtoIX and Mg‐ProtoIX monomethylester (Mg‐ProtoIXme) have been proposed as putative retrograde signals (Figure 3), although this proposition has been subject to scrutiny (Larkin 2016).

In *Arabidopsis*, Strand et al. (2003) initially suggested that Mg‐ProtoIX acts as a repressor of *PhANG* expression by recording increased levels of Mg‐ProtoIX in seedlings where chloroplast biogenesis was blocked by norflurazon. The increase was less pronounced in treated *gun2* and *gun5* mutants, which also kept high *PhANG* expression. In subsequent years, a number of different publications provided evidence to support a model whereby Mg‐ProtoIX or Mg‐ProtoIXme downregulates *PhANG* expression (Alawady and Grimm 2005; Ankele et al. 2007). This model was challenged by later studies, which did not record a similar correlation between Mg‐ProtoIX levels and *PhANG* repression (Mochizuki et al. 2008; Moulin et al. 2008).

Some of the mentioned contradictories can be explained by the fact that the accumulation of porphyrin intermediates following stress is short‐lived and transient (Zhang et al. 2011). As persistent accumulation of chlorophyll intermediates results in photodynamic as well as physiological damage (Meskauskiene et al. 2001), the high turnover of Mg‐ProtoIX and Mg‐ProtoIXme could circumvent toxicity. Additionally, it is known that both Mg‐ProtoIX and Mg‐ProtoIXme levels oscillate in a photoperiodic light regime, peaking during the light period and dropping during the dark (Norén et al. 2016). It is therefore hard to rule out the impact of the experimental set‐up on the regulation of *PhANG* expression.

Another proposition about the effect of the *gun5* mutation is that it diverts protoporphyrin IX from Mg‐ProtoIX, towards Fe‐protoporphyrin, thereby increasing haem levels (Woodson et al. 2011) (Figure 2). Indeed, haem can act as a positive biogenic retrograde signal that controls nuclear gene expression. Evidence supporting this arose from the discovery of the *gun6‐1D* mutant that overexpresses *FC1* (*FERROCHELATASE 1*). Akin to the other *GUN* alleles, *gun6‐1D* plants exhibit a derepression of *PhANG* expression under NF (Woodson et al. 2011). This study failed to observe a *gun* phenotype in plants overexpressing a second ferrochelatase isoform (*FC2*), thus indicating that only FC1‐derived haem acts as a retrograde signal. Moreover, in the dark‐grown *Chlamydomonas reinhardtii* cultures, haem feeding induced transcription of the nuclear gene *HEAT SHOCK PROTEIN 70 A* (*HSP70A*), further suggesting haem as a positive regulator of nuclear gene expression (Von Gromoff et al. 2008). Despite *GUN2* encoding haem oxygenase (Figure 2), conflicting data exist relating to whether *gun2* accumulates more or less haem relative to wild‐type plants after NF treatment (Woodson et al. 2011).

One of the primary issues with correlating haem accumulation to *PhANG* expression relates to quantification methods. Haem is associated with many different proteins known as hemoproteins, which carry out vital biological functions. It is generally believed that a non‐specifically bound pool of ‘free’ haem acts as a retrograde signal. Due to poor solubility, it is unlikely that free haem accumulates in the cytosol, but it is more likely present in membranes or bound non‐specifically to proteins (Thomas and Weinstein 1990). Efforts have been made to establish a methodology for distinguishing this pool of free haem from haem associated with hemoproteins (Espinas et al. 2012). Nonetheless, inconsistencies in protocols used in different studies may account for contradictory findings.

Perhaps the strongest evidence favouring haem as a retrograde signal is the fact that it is exported from plastids (Thomas and Weinstein 1990). Moreover, haem has been recognised as a signalling molecule in different types of organisms, among which are yeast (Zhang and Hach 1999) and animals (Bottino‐Rojas et al. 2015). In that regard, many researchers consider haem as the primary tetrapyrrole‐derived mobile retrograde signal arising during chloroplast biogenesis (Larkin 2016).

### 3.2 Plastid Signal Integration via GUN1

The original *gun* mutant screen of Susek et al. (1993) yielded five genes encoding enzymes involved in the tetrapyrrole synthesis pathway, as described above. The sixth candidate from this study is *GUN1*, which encodes a chloroplast‐localised pentatricopeptide repeat (PPR) protein and received particular interest, as it seems to integrate many different signals during plastid stress. The GUN1 protein is specifically active during the early phases of chloroplast biogenesis and destabilises when chloroplasts mature (Hernández‐Verdeja et al. 2022).

Similar to GUN2–6, GUN1 coordinates the flux of the tetrapyrrole synthesis pathway at two different levels: First, it inhibits the pathway via to‐date unknown binding to enzymes or porphyrins, resulting in reduced haem and Pchlide levels after ALA (aminolevulinic acid) feeding in the presence of GUN1 (Shimizu et al. 2019). Second, it promotes FC1 affinity with its substrate, likely by directly interacting with Proto‐IX (Figures 2 and 3). This results in increased haem synthesis in darkness, when GUN1 is active. Due to GUN1's low abundance, these mechanisms will likely not significantly reduce tetrapyrrole levels in darkness, but rapid GUN1 degradation in the light could give the pathway a boost (Shimizu et al. 2019). *Gun1* mutants of *Arabidopsis* maintained *PhANG* gene expression not only after NF but also after lincomycin treatment, which suggests a role in retrograde signalling beyond the tetrapyrrole synthesis pathway.

The PPR domains of the GUN1 protein could interact with nucleic acids. In vitro studies confirmed GUN1 binding to DNA a long time ago, but later work additionally identified a function in RNA editing and stability via interaction with MULTIPLE ORGANELLAR RNA EDITING FACTOR 2 (MORF2) (Zhao et al. 2019). Initially, it was thought that only MORF2 bound to RNA molecules, but recent work revealed that GUN1 can directly interact with and stabilise target transcripts, rRNA and tRNA, as well (Tang et al. 2024).

Besides its role in post‐translational RNA editing, GUN1 steers the plastid‐encoded protein pool at other levels as well. GUN1 regulates mRNA levels, the accumulation of the PLASTID RIBOSOMAL PROTEIN S1 (PRPS1), and interacts with proteins involved in plastid protein homoeostasis (Tadini et al. 2016; Marino et al. 2019). Lastly, by binding to chloroplast HEAT SHOCK PROTEIN70‐1, GUN1 regulates the import of cytosolic (pre‐)proteins into the chloroplast. As a consequence, accumulation of these proteins in the cytosol causes *gun*‐like phenotypes (Wu et al. 2019).

The molecular function of GUN1 seems diverse and interferes with many different plastid pathways that release retrograde signals. This might make GUN1 the most pronounced integrator of retrograde signals upon stress. Indeed, GUN1 seems to be involved in the responses to high light, leading to altered development and increased photoprotection (Martín et al. 2016; Richter et al. 2020).

### 3.3 Signals From the Plastid Transcription and Translational Machinery

Plastid transcription is carried out by two primary RNA polymerases: nuclear‐encoded RNA polymerase (NEP) and plastid‐encoded RNA polymerase (PEP). During chloroplast biogenesis, NEP is the main RNA polymerase in etioplasts, while PEP is the primary regulator of transcription in mature chloroplasts (Hernández‐Verdeja et al. 2020). It is worth noting that, though less active, a fully assembled PEP complex has recently been shown in both proplastids and etioplasts of dark‐grown *Arabidopsis* cell cultures and seedlings, respectively (Ji et al. 2021). It is the general shift in polymerase activity rather than existence that characterises chloroplast biogenesis. This shift is initiated when light is perceived by nuclear photoreceptors (Hernández‐Verdeja et al. 2020). Thus, the initiation of PGE requires anterograde signalling for the full activation of PEP. The use of chemical inhibitors that directly block plastid transcription or translation elegantly showed the role of retrograde signals arising from the plastid protein synthesis machinery in regulating chloroplast development. Upon treatment with inhibitors such as lincomycin, rifampicin or tagetoxin, the expression of various *PhANG*s is downregulated (Rapp and Mullet 1991).

Repression of *PhANG* expression is also present in mutants for genes encoding components of the transcriptional machinery of chloroplasts. One example is the sigma factors, which are required for the correct promotor binding of PEP (Lerbs‐Mache 2011). Especially, mutations in SIG2 and SIG6 lead to a pale‐green leaf phenotype, accompanied by a reduction in *PhANG* and plastid transcription, further suggesting a relationship between plastid and nuclear gene expression (Woodson et al. 2013).

During chloroplast biogenesis, the PEP core forms a complex with 12 nuclear‐encoded PEP‐ASSOCIATED PROTEINs (PAPs), which are required for PEP functionality. Inactivation of the *PAP* genes results in low PEP activity, as well as an albino or pale leaf phenotype, thus confirming the PAPs' involvement in chloroplast development (Pfalz and Pfannschmidt 2013). Interestingly, expression profiling of a *pap7* mutant revealed disturbances in both plastid and nuclear gene expression (Grübler et al. 2017). *Pap7* and *pap8* mutants showed defects in the accumulation of the GOLDEN‐LIKE transcription factors (GLK1 and GLK2), responsible for the expression of nuclear photosynthetic genes, further linking PEP function to nuclear expression (Grübler et al. 2017; Liebers et al. 2020).

### 3.4 Photosynthesis‐Derived Signals

Photosynthesis is a highly sensitive process that requires coordinated *PhANG* and *PhAPG* expression to respond to fluctuations in the environment. Alterations in photosynthesis can induce a variety of retrograde signals (Figure 3). These include those generated directly from the PETC, redox‐active enzymes, ROS and sugars. In general, these signals are induced by disturbances to the photosynthetic redox balance.

Of the different photosynthesis‐derived signalling components, ROS are perhaps the best known. The most common include ^1^O_2_, O_2_^●−^, H_2_O_2_ and ^•^OH. They all possess unique properties, for example, ^•^OH is the most reactive and short‐lived, whereas H_2_O_2_ is the most stable and long‐lived of these four (Mittler 2017). Though long regarded as toxic by‐products of aerobic metabolism, research has revealed that ROS should be maintained at a basal level for optimal plant health (Schieber and Chandel 2014; Mittler 2017). One of the best annotated retrograde signals inducing ROS is ^1^O_2_, on which we will elaborate further in this review.

The first evidence supporting the role of ^1^O_2_ as a retrograde signalling component arose from the identification of the protochlorophyllide (Pchlide) accumulating *fluorescent in blue light* (*flu*) mutant (Meskauskiene et al. 2001). The *flu* mutant offered an ideal system for exploring ROS‐derived stress responses, as Pchlide, accumulating in etioplasts, can transfer light energy to ground‐state oxygen, yielding a ^1^O_2_ burst upon seedling illumination (Op Den Camp et al. 2003). Shortly after a dark‐to‐light shift, *flu* plants exhibit necrotic lesions, as well as increases in ^1^O_2_‐responsive gene (*SORG*) expression (Op Den Camp et al. 2003). These responses may appear to be induced by ^1^O_2_ toxicity, but subsequent studies suggested an alternative hypothesis. Inactivation of *EXECUTER1* (*EX1*) and its homologue *EX2* abolished the necrotic phenotype of *flu*, despite maintaining elevated ^1^O_2_ (Lee et al. 2007). Moreover, the upregulation of *SORG* expression in *flu* is abolished in an *ex1 ex2 flu* triple mutant, providing evidence for *EX1* and *EX2* as active ^1^O_2_ signalling components (Lee et al. 2007). These findings provided evidence for a retrograde signalling system that controls necrosis in response to ^1^O_2_ elevation in plastids.

EX1 is localised to the chloroplast grana margins, in the same region where chlorophyll synthesis and PSII repair take place (Wang et al. 2016). PSII repair is carried out by an ATP‐dependent zinc metalloprotease, FtsH (Kato and Sakamoto 2018). Moreover, degradation of EX1 shortly following light exposure appears to be dependent on FtsH, suggesting EX1 may be a target of proteolysis (Dogra et al. 2017; Wang et al. 2016). FtsH inactivation in *flu* background led to a strong downregulation of ^1^O_2_‐responsive genes, similar to that of the *ex1 flu* mutant. A later study showed that EX1 is a target of oxidation by ^1^O_2_, which primes EX1 for FtsH degradation (Dogra et al. 2019). A recent paper revealed that EX2 also undergoes ^1^O_2_‐dependent oxidation and FtsH proteolysis, which in turn further stimulates EX1 oxidation, offering the first evidence for the nature of the EX2–EX1 relationship (Dogra et al. 2022).

Multiple lines of evidence have revealed EX1 and EX2 as regulators of biotic and abiotic stress responses following ^1^O_2_ accumulation. For example, treatment of *Arabidopsis* seedlings with the toxin tenuazonic acid (TeA), isolated from the fungal pathogen *Alternaria alternata,* resulted in an increase in ^1^O_2_, EX1/EX2‐mediated gene expression. TeA blocks the electron transport chain, which leads to an over‐accumulation of reduced plastoquinone (PQ) and the excitation of chlorophyll to a triplet state (^3^Chl) (Chen et al. 2015). In a similar manner, high light stress generates ^3^Chl (Krieger‐Liszkay 2005). With that in mind, it is unsurprising that light stress‐induced photoinhibition was diminished in *ex1 ex2* double mutants (Kim et al. 2012). Finally, a recent study found that mutating *ex1 ex2* completely rescues the high‐light‐induced photobleaching phenotype of de‐etiolating *pif1 pif3* double mutants (Li et al. 2023). EX1 interacts with GUN4 and GUN5 in etioplasts, upon light exposure and the release of ^1^O_2_. Initially, Li et al. (2023) proposed migration of EX1 to the nucleus following ^1^O_2_ exposure; however, this theory has since been rebutted by Liu et al. (2025). Instead, it was suggested that EX1 adheres to the outside of the nucleus during cell fractionation due to methodology‐induced chloroplast rupture (Liu et al. 2025). Nonetheless, these findings further demonstrate the light‐responsive signalling potential of ^1^O_2_.

Whilst ROS are one source of signals arising from altered redox balance during photosynthesis, numerous other components of the photosynthetic redox regulatory system can be impacted by environmental changes. As light drives electron transport during photosynthesis, altered redox homoeostasis can act as an important signal conferring information about light quality to the nucleus. One of the best‐known redox‐active compounds that has been linked to retrograde signalling is PQ (Pfannschmidt et al. 2003).

PQ carries out a range of functions within the chloroplast, including electron transfer from PSII to cytochrome *b*_*6*_ *f* (Havaux 2020), cyclic electron flow around PSI (Johnson 2011), and facilitating the light‐driven reduction of oxygen at PSII to H_2_O_2_ via O_2_^●−^ (Vetoshkina et al. 2017). PQ localises to a range of chloroplast lipid structures (Ksas et al. 2018), but is largely found in thylakoid membranes where it associates with PSII (Havaux 2020). Here, each PSII reaction centre is associated with approximately 5–15 PQ molecules, coining the term ‘plastoquinone pool’ to describe the collective dynamics of these molecules. During electron transport, PQ reduces to PQH_2_ (plastoquinol) molecules. PQH_2_ later releases protons and electrons to return to its oxidised state, allowing fluid electron transport (Havaux 2020). In theory, the ratio of PQH_2_ to PQ represents a putative redox sensor that can transmit information relating to photosynthetic electron transport to downstream (retrograde) signalling pathways (Figure 3).

This proposition was tested for some time, and indeed, the redox state of the PQ pool has been associated with changes in *PhANG* and *PhAPG* expression. A study in *Dunaliella tertiolecta* found that increased *LHCB1* expression, when shifting from high to low light conditions, depends on PQ redox state (Escoubas et al. 1995). Blocking electron transport from PSII to PQ by DCMU (3‐[3,4‐dichlorophenyl]‐1,1‐dimethylurea) under high light led to a partial increase in *LHCB* expression. Similarly, the inhibition of PQH_2_ oxidation by DBMIB (2,5‐dibromo‐3‐methyl‐6‐isopropyl‐*p*‐benzoquinone) in low light induced a higher‐light‐adjusted transcriptional response for *LHCB1* (Escoubas et al. 1995). The redox state of PQ is not only impacted by shifts in light intensity; however, unbalanced excitation of PSII and PSI (e.g., by shifts in the light spectrum) can likewise have an effect (Tullberg et al. 2000). A recent study found the redox state of PQ to regulate stomatal development by altering the expression of *SPEECHLESS* and *MUTE* transcription factors, which directly links photosynthesis‐derived retrograde signals to physiological adaptation (Zoulias et al. 2021).

Akin to many sources of retrograde signals, the nature of signal transmission from PQ remains elusive. One putative signalling component of the PQ redox state is the serine/threonine protein kinase STATE TRANSITION 7 (STN7), a known regulator of LHCII phosphorylation within PSII (Bellafiore et al. 2005). Over‐excitation of PSII relative to PSI leads to PQ reduction, activating STN7, in a manner that is dependent on the docking of PQH_2_ within the Qo site of the Cyt *b*_6_ *f* complex (Zito 1999). Active STN7 phosphorylates LHCB1 and LHCB2, leading to their dissociation from PSII (Lemeille et al. 2009), as well as the relocation of primarily LHCB2 to PSI, thus altering photosystem antenna sizes (Longoni et al. 2015). Mutants lacking functional STN7 fail to perform this state transition (Bellafiore et al. 2005), whilst also exhibiting larger grana diameter and reduced stacking dynamics under different light intensities (Hepworth et al. 2021; Flannery et al. 2023). While STN7's role in regulating phosphorylation is local, changes in thylakoid dynamics suggest a more distant impact on nuclear transcription.

### 3.5 Non‐Tetrapyrrole‐Derived Metabolic Signals

Plastids are metabolic factories that drive a myriad of biochemical processes, including the synthesis of fatty acids, plant hormones, vitamins and secondary metabolites. As with photosynthesis, plastid primary and secondary metabolism is governed by nuclear gene expression, but is impacted by stressors, thus requiring retrograde signalling to remain in balance. The retrograde signals arising from altered non‐photosynthetic tetrapyrrole‐based metabolism have been studied primarily in the context of operational control, with 2‐C‐Methyl‐d‐erythritol‐2,4‐cyclophosphate (MEcPP), various carotenoid derivatives and 3′‐phosphoadenosine 5′‐phosphate (PAP) being the best known signalling metabolites (Jiang and Dehesh 2021).

Many of the known plastid metabolites implicated in retrograde signalling arise from the methylerythritol phosphate (MEP) pathway—an evolutionarily conserved, indispensable pathway present in all plastid‐bearing organisms (Zeng and Dehesh 2021). This pathway drives the production of the functionally and structurally diverse class of metabolites known as the isoprenoids (Allamand et al. 2023), which include plant hormones, pigments and various secondary metabolites (Rodríguez‐Concepción 2014) (Figure 2). One intermediate of the MEP pathway, MEcPP (Figures 2 and 3), was revealed as a retrograde signalling molecule resulting from a genetic screen for regulators of the stress‐inducible, nuclear‐encoded gene *HYDROPEROXIDE LYASE* (*HPL*) (Xiao et al. 2012). An amino acid substitution in the gene encoding HYDROXY‐2‐METHYL‐2‐(E)‐BUTENYL 4‐DIPHOSPHATE SYNTHASE (HDS) generated an MEcPP accumulating mutant (*ceh1*), which was characterised by a strong transcriptional upregulation of not only *HPL*, but also *ARABIDOPSIS ISOCHORISMATE SYNTHASE 1* (*ICS1*). *ceh1* accumulates higher levels of salicylic acid (SA), which in turn confers enhanced resistance to infection by *Pseudomonas syringae* (Xiao et al. 2012). In wild‐type plants, MEcPP levels increase in response to stressors such as wounding, pathogen attack and high light (Xiao et al. 2012; Wang et al. 2017; Onkokesung et al. 2019), suggesting its role in stress signalling.

Following accumulation, MEcPP can elicit pleiotropic responses by regulating the transcription of a variety of nuclear‐encoded genes. In spite of this, the exact mechanism by which MEcPP activates stress‐responsive gene expression remains elusive. One study found that MEcPP activated the transcription factor CALMODULIN‐BINDING TRANSCRIPTION ACTIVATOR 3 (CAMTA3) in a Ca^2+^ manner, although the exact mechanism of CAMTA3 induction is unclear (Benn et al. 2016). Interestingly, MEcPP also accumulates in bacteria in response to oxidative stress (Ostrovsky et al. 1998). In *Chlamydia trachomatis*, MEcPP has been implicated in chromatin remodelling by disrupting the activity of Histone H1‐like protein (Hc1) required for maintaining genome compaction (Grieshaber et al. 2004). Such studies in bacteria may provide insight into the mechanism of regulation in plants, but should be investigated cautiously due to the complexity of signalling between organelles.

Another set of MEP pathway‐derived metabolites involved in retrograde signalling are the carotenoid derivatives (Figures 2 and 3). Carotenoids are a diverse group of isoprenoid metabolites that play vital roles in photosynthesis and photoprotection (Sun et al. 2022). They are considered to be the main quenchers of ^1^O_2_ in the plastids. The role of carotenoids in retrograde signalling is also revealed through the extensive use of NF as a means to alter *PhANG* expression (Figure 3). NF inhibits PHYTOENE DESATURASE (PDS), which converts phytoene to ζ‐carotene, leading to extensive photooxidation and bleaching of seedlings in light (Foudree et al. 2010) (Figure 2). The role of carotenoids in retrograde signalling spans beyond their antioxidative properties; however, a large body of research has suggested that the carotenoids themselves regulate nuclear gene expression.

β‐Cyclocitral (β‐CC), a volatile apocarotenoid generated through the ^1^O_2_‐mediated oxidation of β‐carotene (Ramel et al. 2012), is one well‐studied proposed retrograde signalling molecule (Figure 2). Under high‐light conditions, β‐CC acts by promoting SA synthesis, resulting in the nuclear localisation of NONEXPRESSOR OF PATHOGENESIS‐RELATED GENES 1 (NPR1) and subsequent transcriptional reprogramming of ROS detoxification genes, thereby enhancing plant tolerance to oxidative stress (Lv et al. 2015). Another study found protective effects of β‐CC treatment under cold conditions, which was likewise attributed to changes in stress‐related gene expression (Ramel et al. 2012). Later, it was shown that the resistance of young leaves to high light depended on β‐CC‐mediated *SCARECROW LIKE14* (*SCL14*) upregulation and the induction of associated reactive carbonyl species (RCS) detoxification (D'Alessandro et al. 2018). Furthermore, β‐CC improves drought and salt tolerance, possibly by changing root development (D'Alessandro et al. 2019; Dickinson et al. 2019; Braat et al. 2023). However, the exact nature of β‐CC signalling warrants further investigation.

In addition to β‐CC, other *cis*‐carotene‐derived apocarotenoids also constitute retrograde signalling responses at both biogenic and operational levels. However, in many cases, the exact apocarotenoid responsible for the retrograde signal remains unknown. For example, mutating the *ZDS/CHLOROPLAST BIOGENESIS5 (CLB5)* gene induces alterations in *PhAPG* and *PhANG* expression, accompanied by albinism and abnormal leaf morphology, through the action of an unknown apocarotenoid (Avendaño‐Vázquez et al. 2014). Similarly, the post‐transcriptional regulation of PSY, the enzyme catalysing the first committed step of *cis*‐carotene biosynthesis (Figure 2), was proposed to be regulated by plastid‐derived apocarotenoid levels (Alvarez et al. 2016). Another well‐characterised carotenoid biosynthesis mutant is the Arabidopsis *carotenoid and chloroplast regulation 2* (*ccr2*) mutant, affected in the gene encoding the enzyme CAROTENOID ISOMERASE (CRTISO; Figure 2). Etioplasts of dark‐grown *ccr2* seedlings lack prolamellar bodies, resulting in perturbed photosynthesis establishment after transitioning to light (Park et al. 2002). This phenotype was attributed to another, undefined, *cis*‐carotene‐derived apocarotenoid that accumulates under dark or short‐day conditions (Cazzonelli et al. 2020). Such an apocarotenoid must be synthesised from enzymatic steps upstream of CRTISO. Indeed, second mutations in either *ζ‐CAROTENE ISOMERASE* (Cazzonelli et al. 2020) or *PSY* rescue plastid abnormalities in *ccr2*, possibly by altering nuclear transcription of core photomorphogenesis components such as the *PIFs* and *ELONGATED HYPOCOTYL 5* (Cazzonelli et al. 2020; Hou et al. 2024).

Whilst it is clear that altering enzymatic steps of the apocarotenoid biosynthesis pathway has implications for plastid development and nuclear gene expression, further research is needed to better describe the nature of these signals. Whether these apocarotenoids are indeed retrograde signals that are exported from the plastids to regulate nuclear gene expression remains unclear. One may consider the possibility that overaccumulation of specific *cis‐*apocarotenoids has direct impacts on plastid ultrastructure, which has concomitant effects on nuclear gene expression through unknown signalling pathways. Taken together, this field offers an exciting opportunity for future research in operational retrograde signalling.

One other metabolism‐derived retrograde signal source is that of PAP and the nucleotide phosphatase SAL1 (Figure 3). PAP is generated as a by‐product of sulphur metabolism, but is degraded by SAL1 under normal growth conditions (Chen et al. 2011; Estavillo et al. 2011). Interestingly, early research on SAL1 identified its role in regulating stress responses using a genetic screen for mutants with upregulated expression of the antioxidant *ASCORBATE PEROXIDASE 2* (Rossel et al. 2006). This *altered expression of APX2* (*alx8*) mutant holds a null mutation in *SAL1*, which provides resistance to high light and drought stress (Rossel et al. 2006; Wilson et al. 2009). Such tolerance was later attributed to an accumulation of plastid‐localised PAP (Estavillo et al. 2011). The SAL1‐PAP module, therefore, presented itself as a putative stress‐responsive retrograde signalling module. High light and drought stress induce oxidative stress within the plastids, offering a clue to the regulation of the SAL1‐PAP pathway. Increasing plastid oxidative stress reduces SAL1 activity through dimerisation, disulphide bond formation and glutathionylation (Chan et al. 2016a). This presents SAL1's role outside of sulphur metabolism, as an oxidative stress sensor that regulates the expression of plastid redox‐associated nuclear genes (such as *APX2*) via PAP.

Under heat stress, PAP accumulates as a result of increased tocopherol levels. In this case, the PAP induction is independent of oxidative stress and SAL1 and induces nuclear miRNAs that promote heat tolerance (Fang et al. 2019). The fact that PAP translocates from the cytosol to plastids (Bohrer et al. 2014) makes it a good candidate for intracellular signalling activity.

## 4 Stressed Chloroplasts Steer Plant Development Through Retrograde Signalling

So far, we have described how various abiotic conditions cause chloroplast stress and the retrograde signals emitted from stressed chloroplasts that affect nuclear gene expression and stress tolerance. The effects of retrograde signals on stress resilience are often studied using chloroplast stress‐inducing antibiotics or mutants that display constitutive retrograde signalling. Nevertheless, a number of studies have shown that abiotic stress induces retrograde signalling through GUN1, MEcPP, carotenoid derivatives and SAL1‐PAP. In addition to the regulation of *PhANG* or stress‐responsive gene expression, these chloroplast‐derived signals can strongly affect whole plant physiology, growth and development by interfering with canonical signalling pathways.

### 4.1 Retrograde Signals Interact With Photoreceptor Signalling

Plants constantly monitor their light environment and adapt their growth to best suit the local conditions. To this end, they possess dedicated wavelength‐specific photoreceptors that together sense most of the light spectrum. Depletion of photosynthetically important blue and red light in darkness or shaded environments leads to inactivation of cryptochrome (cry) and phytochrome (phy) photoreceptors, respectively. This photoreceptor inactivation releases the repression of PIFs, resulting in auxin synthesis and shoot elongation as well as an inhibition of photomorphogenesis. In light, on the other hand, activated cry and phy repress PIFs and inhibit the CONSTITUTIVELY PHOTOMORPHOGENIC 1 (COP1)/SUPPRESSOR OF PHYTOCHROME A‐105 (SPA) E3 ubiquitin ligase complex, which results in stabilisation of the photomorphogenesis‐promoting transcription factor HY5 (Gommers and Monte 2017). Among the HY5‐induced genes are the *GOLDEN 2‐LIKE* (*GLK*) transcription factors *GLK1* and *GLK2*, which are oppositely regulated in darkness by repressive PIF binding (Zhang et al. 2024; Martín et al. 2016). These GLKs promote photomorphogenesis by direct stimulation of *PhANG* expression as well as through enhanced expression of the transcription factor *B‐BOX16* (Veciana et al. 2022).

Among the first identified common targets of light and retrograde signalling are cry1 and HY5. A screen for *gun* mutants revealed that, like *gun1*, *cry1* mutants maintain partial light‐induced *LHCB1.1* expression in the presence of chloroplast‐inhibiting antibiotics (Ruckle et al. 2007). Mutation of *cry1* or *hy5* in a *gun1* background further increased *LHCB1.1* expression in light with lincomycin, suggesting additive effects for GUN1, cry1 and HY5 in retrograde signalling. Moreover, these mutations severely increased photobleaching in high light, especially in higher‐order mutants, suggesting that retrograde or photoreceptor signalling through these proteins limits photooxidative damage (Ruckle et al. 2007). Later studies showed that GUN1, cry1, HY5 and GLK2 are required for anthocyanin synthesis, to reduce photooxidative damage in high light (Richter et al. 2023).

Retrograde and photoreceptor signalling interact downstream of phy signalling as well. In light, phy represses PIFs and thereby inhibits the repressive effect of PIFs on *GLK1* expression (Martín et al. 2016). Similarly, *GLK1* expression is repressed under stressfully high light conditions or after lincomycin treatment. This suppression is mediated by GUN1 and is independent of PIFs and HY5 (Martín et al. 2016), suggesting that the phy and GUN1 signalling pathways antagonistically regulate *GLK1* expression. Interestingly, the repression of *GLK1* leads to the inhibition of seedling photomorphogenesis at multiple scales. Besides the suppression of *PhANG* expression, it prevents the opening and expansion of cotyledons in high light, resulting in a mild skotomorphogenic phenotype (Martín et al. 2016; Veciana et al. 2022). This impact on seedling development is GUN1‐dependent and the result of the inhibition of both *GLK1* expression and GLK1‐induced *BBX16* expression (Veciana et al. 2022). As a consequence, the overexpression of either *GLK1* or *BBX16* results in a strongly reduced response to lincomycin or high light, which is largely restored by the *bbx16* mutation in *GLK1OX*.

In contrast to GUN1‐mediated retrograde signalling, the isoprenoid precursor MEcPP affects phy signalling more upstream. As high light causes accumulation of various stress compounds besides MEcPP, its effects are mostly studied by exogenous application or using MEcPP‐accumulating *ceh1* mutants (Xiao et al. 2012). These *ceh1* mutants have short hypocotyls in light, which could be explained by phyB stabilisation and reduced PIF activity (Jiang et al. 2019). Next to high light, MEcPP accumulates in response to high temperature (Rivasseau et al. 2009). Warm temperatures induce a developmental programme named thermomorphogenesis, which shares many key signalling components with light responses, including phyB and PIFs, so it is likely that MEcPP‐induced retrograde signalling influences this developmental programme as well.

The aforementioned ccr2 mutant, lacking the CRTISO enzyme (Figure 2), accumulates *cis*‐carotenoids in plants that are shifted from long to short photoperiods (Cazzonelli et al. 2020). These *cis*‐carotenoids are cleaved to apocarotenoids, introducing a retrograde apocarotenoid signal that inhibits *HY5* and *PhANG* expression, while inducing *PIF3* expression (Cazzonelli et al. 2020). The resulting impact on gene expression impairs chloroplast biogenesis and photomorphogenesis, thereby linking another retrograde signal to photoreceptor signalling.

### 4.2 Retrograde Signals Interact With Hormone Signalling

Phytohormone signalling pathways are sensitive to disruption by stress‐induced retrograde signalling as well. This is perhaps unsurprising, as the previously discussed photoreceptor pathways rely on the synthesis and action of various growth‐related hormones. However, retrograde signalling also affects hormonal pathways that are not directly associated with photoreceptors, indicating a broader impact on growth and stress responses.

As previously mentioned, MEcPP accumulation reduces hypocotyl growth through enhanced phyB abundance and reduced PIF activity. In addition to this, MEcPP also disrupts the abundance and localisation of PIN auxin transporters and reduces the expression of the YUCCA auxin synthesis genes (Jiang et al. 2018; Jiang et al. 2020). As a consequence, MEcPP reduces auxin‐mediated hypocotyl elongation, as visualised using the DR5::GFP auxin signalling marker. Strikingly, DR5::GFP abundance in the hypocotyl is similarly affected in high light, suggesting biological relevance of this chloroplast‐to‐nucleus signalling pathway beyond the MEcPP accumulating *ceh1* mutant or exogenous treatment (Jiang et al. 2018).

In addition to auxin, MEcPP accumulation reduces ethylene abundance, probably through reduced expression of various ACC SYNTHASE (ACS) genes that facilitate the production of the ethylene precursor 1‐aminocyclopropane‐1‐carboxylate (ACC) (Jiang et al. 2020). ACC treatment partially rescues hypocotyl length in dwarfed *ceh1* plants, but only in seedlings that retain normal auxin signalling, suggesting ethylene is epistatic to auxin in the regulation of hypocotyl elongation (Jiang et al. 2020). Besides hypocotyl elongation, ethylene signalling mediates the lincomycin‐induced inhibition of cotyledon separation in light (Gommers et al. 2020). Although the lincomycin‐induced retrograde signal that targets the ethylene signalling pathway remains elusive, it is GUN1‐independent (Gommers et al. 2020).

The oxidative stress that occurs during high light and drought causes chloroplast accumulation of PAP, through inactivation of the PAP‐degrading enzyme SAL1. Mutation of *sal1* in Arabidopsis causes various defects in development and growth, including altered venation, short hypocotyls, compact rosette structure, delayed circadian rhythmicity and disturbed flowering (reviewed in Phua et al. [2018]). These phenotypic defects were identified in a range of forward genetics studies, which gave rise to a plethora of described mutant alleles and at least eight different mutant names, further demonstrating the developmental importance of SAL1 and PAP (Phua et al. 2018). In addition to the onset of specific stress‐responsive genes, PAP accumulation affects the expression levels of ABA, auxin, gibberellin and JA biosynthesis genes, many of which are upregulated (Wilson et al. 2009; Estavillo et al. 2011; Phua et al. 2018). Consequently, changes in hormone levels have been found in 4‐week‐old Arabidopsis *sal1* mutants, with reported increases in ABA (Rossel et al. 2006), auxin (Phua et al. 2018) and JA (Rodríguez et al. 2010), but decreased gibberellin levels (Phua et al. 2018).

### 4.3 Carotenoid Derivatives Modulate Root Development

Besides the previously described effects of β‐CC on *SCL14* expression and RCS detoxification, exogenous β‐CC application enhanced root growth in *Arabidopsis*, rice and tomato (Dickinson et al. 2019). Moreover, β‐CC improves the growth of both the root and shoot of salt‐stressed rice. A study that was published around the same time revealed that β‐CC is converted to water‐soluble β‐cyclocitric acid (β‐CCA) in vivo, which similarly promotes expression of β‐CC‐responsive genes (D'Alessandro et al. 2019). In this study, β‐CCA was shown to accumulate during drought and promote drought tolerance and root growth. In drought‐treated tomato, exogenous β‐CCA application reduced water loss and increased total fruit weight (D'Alessandro et al. 2019).

Although these findings suggest that apocarotenoids increase drought tolerance by enhancing root growth, a later study found a repressive effect of both apocarotenoids on root size, while maintaining the enhanced drought tolerance (Braat et al. 2023). This could be the result of β‐CCA‐induced root suberisation, which forms a water diffusion barrier in the root endodermis and reduces water loss. While these studies appear to be contradictory regarding root growth, the effect on drought tolerance is maintained between them.

## 5 Perspectives and Conclusions

In this review, we characterised known retrograde signals and their interaction with canonical signalling pathways. While these interactions provide a solution for improving plant stress adaptation, many studies focus on signalling in the context of biogenic control. In terms of high light stress, photomorphogenesis and the initiation of photosynthesis introduce the risk of photo‐oxidative damage. Retrograde signals released in high light mitigate this stress‐induced damage (Jiang and Dehesh 2021). Developmental adaptations such as unexpanded cotyledons are also thought to serve as a protection of the apical meristem of young seedlings (Martín et al. 2016). However, it remains unknown if this really contributes to higher survival.

Another point of interest is that the extent to which metabolic retrograde signalling pathways are simultaneously induced and interact with one another is not really known. Most studies have focussed on individual pathways; however, recent evidence reveals a far more complex and interconnected scenario. This makes it difficult to identify the source of some retrograde signals. For example, recent studies have identified a number of mutations other than *flu* that can alter ^1^O_2_ production in seedlings. To make matters more complex, the MEP pathway can also generate ^1^O_2_ following light exposure due to imbalanced isoprenoid and tetrapyrrole flux during chlorophyll synthesis (Kim et al. 2013). Since many abiotic stress conditions alter MEP pathway activity (Rivasseau et al. 2009), the possible involvement of ROS‐derived signalling from this pathway should also be considered.

Another challenge in determining the relevance of described signals under natural conditions lies in the way they have been induced. The use of mutants that over‐accumulate retrograde signals (*flu*, *ceh1*, *sal1* and *ccr2* to name a few), or antibiotics that disrupt plastid functionality at different levels, may trigger signalling cascades that differ significantly from those induced by abiotic stress. Whilst such scenarios have provided necessary insight into the signalling role of plastids, they may not fully reflect the complexity or dynamics of natural environments. Advances in metabolomics may provide a means to find new connections between stress‐induced metabolic changes and *PhANG/SRG* expression. These tools may enable a more comprehensive understanding of shifts in plastid metabolism in response to abiotic stress and help bridge the gap between experimental models and real‐world conditions.

Finally, this review started by documenting changes in plastid ultrastructure following abiotic stress exposure (Figure 1). At present, it remains unclear to what extent retrograde signals are implicated in these changes. It is well established that stressed plastids generate retrograde signals and that plastid‐induced reprogramming of nuclear transcription promotes the ultrastructural changes that drive chloroplast biogenesis. With this in mind, it seems likely that stress‐responsive ultrastructural changes require coordinated communication between the plastids and the nucleus. This knowledge gap warrants further investigation and offers an insightful new avenue of research into cellular responses to abiotic stress in plants.

## Conflicts of Interest

The authors declare no conflicts of interest.

## Data Availability

Data sharing is not applicable to this article as no new data were created or analysed in this study.
